# Bacterial and Fungal Biocontrol Agents for Plant Disease Protection: Journey from Lab to Field, Current Status, Challenges, and Global Perspectives

**DOI:** 10.3390/molecules28186735

**Published:** 2023-09-21

**Authors:** Muhammad Ayaz, Cai-Hong Li, Qurban Ali, Wei Zhao, Yuan-Kai Chi, Muhammad Shafiq, Farman Ali, Xi-Yue Yu, Qing Yu, Jing-Tian Zhao, Jing-Wen Yu, Ren-De Qi, Wen-Kun Huang

**Affiliations:** 1 Institute of Plant Protection and Agro-Products Safety, Anhui Academy of Agricultural Sciences, Hefei 230041, China; m.ayazbiotech@aaas.org.cn (M.A.); bioplay@sina.com (W.Z.); chi2005112@163.com (Y.-K.C.); 2 State Key Laboratory for Biology of Plant Diseases and Insect Pests, Institute of Plant Protection, Chinese Academy of Agricultural Sciences, Beijing 100193, China; yuxiyue22@163.com (X.-Y.Y.); yuqing08211994@163.com (Q.Y.); jintianzhao2023@163.com (J.-T.Z.); jingwenyu1996@163.com (J.-W.Y.); 3 Cotton Sciences Research Institute of Hunan, Changde 415101, China; mkslch2013@163.com; 4 Key Laboratory of Integrated Management of Crop Diseases and Pests, Department of Plant Pathology, College of Plant Protection, Nanjing Agricultural University, Nanjing 210095, China; 2020202068@stu.njau.edu.cn; 5 Biology Department and Institute of Marine Sciences, College of Science, Shantou University, Shantou 515063, China; mshafiq@stu.edu.cn; 6 Department of Entomology, Abdul Wali Khan University, Mardan 23200, Pakistan; drfarman@awkum.edu.pk

**Keywords:** biocontrol, natural products, rhizosphere microbes, plant diseases, sustainable agriculture

## Abstract

Plants are constantly exposed to various phytopathogens such as fungi, Oomycetes, nematodes, bacteria, and viruses. These pathogens can significantly reduce the productivity of important crops worldwide, with annual crop yield losses ranging from 20% to 40% caused by various pathogenic diseases. While the use of chemical pesticides has been effective at controlling multiple diseases in major crops, excessive use of synthetic chemicals has detrimental effects on the environment and human health, which discourages pesticide application in the agriculture sector. As a result, researchers worldwide have shifted their focus towards alternative eco-friendly strategies to prevent plant diseases. Biocontrol of phytopathogens is a less toxic and safer method that reduces the severity of various crop diseases. A variety of biological control agents (BCAs) are available for use, but further research is needed to identify potential microbes and their natural products with a broad-spectrum antagonistic activity to control crop diseases. This review aims to highlight the importance of biocontrol strategies for managing crop diseases. Furthermore, the role of beneficial microbes in controlling plant diseases and the current status of their biocontrol mechanisms will be summarized. The review will also cover the challenges and the need for the future development of biocontrol methods to ensure efficient crop disease management for sustainable agriculture.

## 1. Introduction

Phytopathogens pose a serious threat to crop productivity worldwide. To provide adequate food for the world’s growing population, an efficient management system is required to control various crop diseases [1,2]. To date, the development of disease-resistant crops, the application of chemical pesticides, and implementing effective strategies are the primary approaches for controlling plant diseases. These approaches have been instrumental to increase crop output and quality over the past few decades [3]. However, the excessive use of chemical pesticides has resulted in environmental pollution, thereby limiting their use in the agricultural sector [3,4]. Currently, researchers are exploring the use of beneficial microorganisms as an eco-friendly strategy to control crop diseases [3,5]. A diverse range of bacterial genera have demonstrated great potential as biocontrol agents for various plant diseases. Fungi have also been shown to play a significant role in preventing important diseases in major crops [5,6]. Research on fungal strains as biocontrol agents for plant disease control has also received considerable attention. These fungi-based biocontrol agents exhibit a significant antagonistic activity against a variety of soil and airborne plant pathogens, making them potential biopesticides for field or greenhouse studies [5,7].

Biocontrol agents employ diverse mechanisms to protect plants against pathogenic invasion. Using one or a combination of processes, they may interact with the pathogen directly or indirectly to reduce plant disease [1,2]. BCA in the rhizosphere region competes for space and resources, and interferes with pathogens’ pathogenicity through different substances such as lipopeptides, biosurfactants, bacteriocins, volatiles, and enzymes that have antimicrobial effects by slowing the development or metabolic activity of pathogens [8]. BCA could disrupt pathogens’ quorum sensing (QS) by inhibiting the production of signal molecules that launch infections. One example is producing QS inhibitors that can break down QS signal molecules, such as chitinases, pectinases, and lactonases. These inhibitors prevent pathogen invasion and lead to reduced plant disease symptoms [9]. In addition to direct interactions, BCAs may protect plants indirectly by triggering a defense response or promoting plant growth [1,5]. As a result, the host undergoes a wide range of biochemical and molecular defensive processes that serve as a defense mechanism against various pathogens. Furthermore, beneficial microbes may promote plant growth by enhancing nutrients and water uptake or by producing chemicals such as hormones for maintaining plant fitness [7,10]. Many processes take part in the complex interactions between pathogens, beneficial microbes, and plants. Therefore, identifying the mechanisms responsible for biocontrol is a great challenge [1,10]. Hence, understanding the mechanism of action behind a BCA’s protective effect will make it easier to optimize biological control; establish ideal conditions for the interaction between the BCA, the pathogen, and the host; and develop suitable formulations and application techniques to improve plant health and sustainable agriculture.

Beneficial microbes, primarily bacteria and fungi, are abundant sources of various natural compounds that have the potential to control plant diseases at various levels. Microbes that suppress plant pathogens produce natural compounds, including secondary metabolites (SMs) [2,5]. These compounds are structurally diverse and low-molecular-weight compounds that are not essential for survival unless microbes are exposed to unfavorable conditions [11]. SMs, such as antibiotics, toxins, ribosomal peptides (RPs), non-ribosomal peptides (NRPs), polyketides (PKs), and volatile organic compounds (VOCs), are widely reported to possess an antagonistic activity against a variety of plant pathogens [1,12]. These compounds play a significant role in combating various diseases by establishing plant−microbe interactions in the soil system. Certain beneficial microorganisms produce SMs that stimulate plant defense responses and trigger systemic resistance against invading pathogens [2,13]. According to biochemical and genomic investigations, SM genes linked to biocontrol activity have been found to occur in gene clusters [3,14]. The development of various omics techniques, such as whole-genome mining and genome bio-engineering, will help uncover these genes responsible for novel secondary metabolites in different microbes [11,15]. Hence, in-depth research on the fewer aspects of biocontrol strategies will provide a bright future in the agriculture sector. This review will provide sufficient information regarding the current status and future development of biocontrol strategies for sustainable agriculture. We address the advancements that have been made in the prospecting biocontrol strategy, factors affecting the biocontrol agents, and challenges with a focus on legislative procedures and parameters that influence their use for commercialization development. Various bacterial and fungal biocontrol agents against plant pathogens are listed in Table 1.

## 2. Beneficial Bacteria and Crop Disease Management

Many studies have investigated the role of beneficial bacteria in promoting plant growth and disease resistance in crops. At present, many bacteria from various genera, including *Bacillus*, *Paenibacillus*, *Agrobacterium*, *Bradyrhizobium*, *Acinetobacter*, *Azospirillum*, *Azotobacter*, *Pseudomonas*, *Rhizobium* and *Streptomyces,* have been documented as biocontrol agents to control various diseases in major crops [2,4]. Phytopathogens, mainly bacteria, fungi, and nematodes, cause serious plant diseases, which present a significant barrier to sustainable crop health and yield [37]. Crops are highly affected when infected by pathogenic bacteria such as *Pseudomonas savastanoi*, *Xanthomonas axonopodis*, and *Ralstonia solanacearum*, although inoculation with *Bacillus* spp. suppresses pathogen growth and reduces disease severity in many infected crops [4,37]. The use of beneficial bacteria can improve plant growth and control various bacterial, fungal, and nematode diseases without harming the environment, as listed in Table 1.

Beneficial bacteria create biofilms and secondary metabolites, such as surfactin, iturin, bacillomycin, and fengycin, which reduce the plant pathogen population by establishing plant−microbial interactions in the rhizosphere region [1,38]. *Bacillus* spp. attach to mycelial cell walls and deform hyphae through the production of extracellular enzymes such as chitosanase, protease, glucanase, and cellulase [5,39]. Lipopeptides, including fengycin, iturin, pumilacidin, mixirin, and surfactin, are antifungal peptides that function against pathogenic fungi in rhizospheres [1,3]. Many bacteria, particularly *Bacillus*, *Pseudomonas,* and *Burkholderia* spp. are known to suppress nematodes in various plants by affecting nematode behavior such as feeding and reproduction [3,40]. Previous studies have shown that biological treatment with *Bacillus* isolates was effective in controlling a root−knot nematode infestation. They have also been reported to reduce nematode populations in infested roots and soil [2,24]. *Bacillus* spp. have also been reported to stimulate induced systemic resistance in plants against various pathogens through increased defense-related enzyme activity, such as polyphenol oxidase, peroxidase, and phenylalanine ammonia-lyase (PAL), as well as root exudates and modifications such as amino acids and polysaccharides [1,41].

The biocontrol bacteria *Bacillus* and *Pseudomonas* spp. have been reported to be effective against various phytopathogens in major crops. The presence of several biosynthetic gene clusters responsible for the synthesis of secondary metabolites were found in *Bacillus velezensis* isolates [1,42]. These chemically varied bioactive metabolites may be used as a resource for the development of new drugs. The recent studies on bioactive substance synthesis, chemical compositions, interesting bioactive gene clusters, and biological applications of *B. velezensis* and related *Bacillus* species will be effective at preventing plant diseases to ensure sustainable agriculture [5,43]. There are numerous uses for antimicrobial compounds made by *B. velezensis* in the management of plant pathogens. *B. velezensis* uses the lipopeptides surfactin, iturin, and fengycin to produce antagonistic effects against *R. solanacearum* and *Fusarium oxysporum* [12,44]. *B. velezensis* QST713 is used industrially as a biocontrol agent to protect *Agaricus bisporus*, an edible mushroom, in a compost micromodel and inhibit *Trichoderma aggressivum* f. *europium*, which causes green mold disease [45]. To carry out their actions, they seem to principally rely on antibiosis and the development of systemic resistance in a variety of plant species [2,46]. Additionally, *B. subtilis* has been identified as a key player in the control of different crop diseases. *B. subtilis* IBFCBF-4 is a potential biocontrol agent for watermelon *Fusarium* wilt. An important example of an exogenous endophyte for preventing banana *Fusarium* wilt in China is the *B. subtilis* R31 strain isolated from *Dendrobium orchid* leaves [47,48]. Thus, extensive research should be conducted to develop efficient formulation technology and to investigate various natural products with the determination of their antimicrobial activity in vitro as well as in the field to manage different crop diseases efficiently.

## 3. Beneficial Fungi and Crop Diseases Management

**Figure 1:**
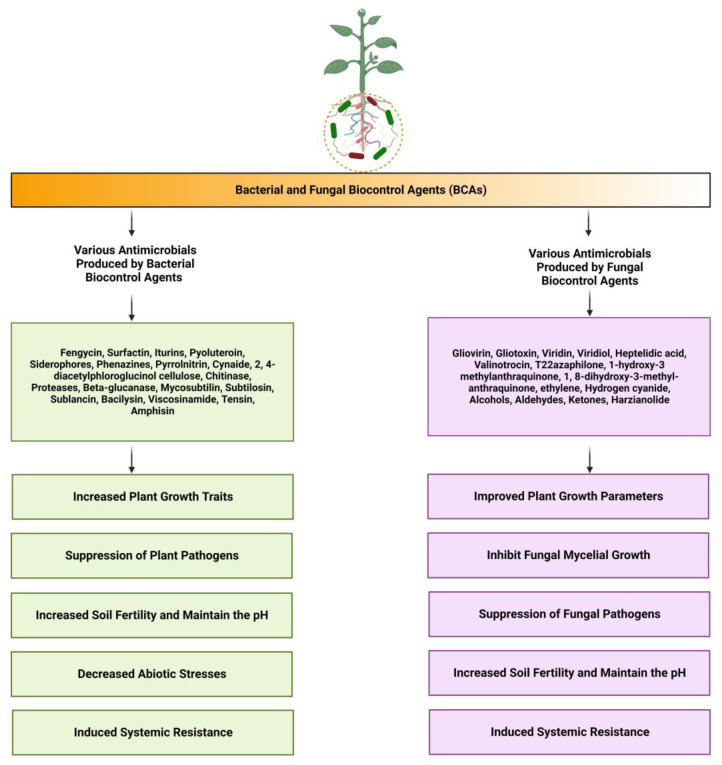
Different antimicrobial products produced by biological control agents, i.e., bacteria and fungi, and their impact on plant pathogens and plant growth parameters. The antimicrobial products produced by bacteria and fungi, such as the secondary metabolites fengycin, surfactin, and bacillomycin D, induce systemic resistance and also have a broad-spectrum antagonistic activity against different diseases. The volatile organic compounds (VOCs) produced by microbes improved plant growth traits and reduced disease indexes, both directly and indirectly. The arrows show the different antimicrobial metabolites produced by bacteria and fungi along with their benefits. The figure was created with BioRender software (https://www.biorender.com/, accessed on 13 September 2023).

Beneficial fungi produce large quantities of bioactive compounds that can be used as agrochemicals for crop protection. The development of fungal strains as BCAs for plant diseases has drawn a lot of attention because it has been found that many beneficial fungi inhibit the growth of various plant pathogens [5,49]. *Trichoderma*, *Aspergillus*, and *Penicillium* are among the most popular fungal genera used as BCAs against both bacterial and fungal plant diseases. Fungi, such as *Gliocladium* and *Saccharomyces,* have also been reported to possess an antagonistic activity against a variety of pathogens [1,50]. Most fungal endophytes, which are diverse microorganisms, live asymptomatically in the internal plant tissues. Endophytic fungi interact closely and intricately with their hosts through mutualism and in rare cases, parasitism [51,52]. They are crucial for protecting their hosts from pathogenic bacteria and pests because of their capacity to produce a large variety of structurally varied and physiologically active secondary metabolites [5,12]. Endophytic fungi are a rich mine for discovering novel secondary metabolites with a wide range of potential agricultural applications [53]. Numerous metabolites with various chemical structures, including terpenoids, alkaloids, steroids, peptides, isocoumarins, benzopyranones, and quinones, have been identified in endophytic fungi. The identification of these metabolites provided a solid chemical basis for the development of agrochemicals that may have antibacterial, antifungal, herbicidal, nematicidal, insecticidal, and other agricultural uses [54,55]. Hence, there is a large variety of fungi that interact with plants in rhizospheric and endophytic associations. Different antimicrobial products produced by biological control agents, i.e., bacteria and fungi, and their impact on plant pathogens along with plant growth parameters are briefly summarized in Figure 1.

It has been discovered that many fungi, especially *Trichoderma*, are famous for their broad-spectrum antagonistic activities against various phytopathogens. *Trichoderma* is a widespread fungal genus found in soil as residents, saprotrophs, plant symbionts, and mycoparasites. This genus contains filamentous fungi that have been extensively investigated and employed as biocontrol agents against plant pathogens in agriculture [56,57]. The direct and indirect control potential of BCAs against phytopathogens has been highly studied in the last decade to ensure efficient plant disease management. It has been demonstrated that *Trichoderma* controls insect pests directly through parasitism and the synthesis of insecticidal compounds, repelling metabolites and antifeedant chemicals. The indirect approaches include attracting natural enemies, stimulating systemic defense mechanisms of plant, and parasitizing insect−symbiotic microbes [46,58]. *Trichoderma* use in agriculture is thus effective at fighting insect pests and plant pathogens, presenting it as a promising future option for the advancement of sustainable agriculture. This fungus has shown a strong antagonistic potential against more than 80% of plant pathogens [49,56]. *T. aggressivum* f. *europaeum* TAET1 showed a strong antagonistic activity against *Botrytis cinerea*, *Sclerotinia sclerotiorum*, and *Mycosphaerella melonis* by affecting mycelial growth. Additionally, it completely stopped *S. sclerotiorum* sclerotia germination [59,60]. *Trichoderma asperellum* TaspHu1, isolated from *Juglans mandshurica* Maxim. rhizosphere soils in China has been shown to enhance resistance in tomato seedlings against leaf spot disease caused by *Alternaria alternata* [61]. It has been clearly shown that *Trichoderma* has a significant role in inhibiting plant pathogens indirectly in the rhizospheric region through enhanced plant immunity. Thus, *Trichoderma* spp. can be employed in combination with several other alternative pest management techniques for sustainable agriculture [49,56].

## 4. Biocontrol Mechanisms in Controlling Plant Diseases

Comprehending the biocontrol mechanisms of plant pathogens is imperative in establishing an optimal environment for effectively managing a diverse range of plant diseases. Many studies have been conducted over the past 20 years related to the role of biocontrol agents in terms of crop disease prevention, rhizosphere colonization, and plant-growth promotion [1,62]. Biocontrol mechanisms of plant diseases mostly include antibiosis, the creation of hydrolytic enzymes, competition for micronutrients, rhizosphere competence, and the development of systemic resistance in host plants [49,63]. Several BCAs have already been demonstrated to trigger induced system resistance (ISR) in various infected crops. Beneficial bacteria such as *Pseudomonas* spp. and *Bacillus* spp. may assist in the development of broad-spectrum disease resistance in plants through enhanced immunity [2,64]. Many studies have shown that beneficial root endophytes, such as *Glomus* spp. and *Trichoderma,* reduce endoparasitic nematode infections by activating the plant immune system [46,46].

The potential of BCAs to manage plant diseases includes different methods. The optimization of biocontrol will be made easier by studying the mechanisms behind the beneficial role of microbes [4,48]. BCAs might use these mechanisms directly or indirectly to combat plant diseases. In a direct approach, BCAs react antagonistically to the pathogen in a direct manner, including antibiosis, parasitism, pathogenicity reduction, and infection, through competing in the rhizosphere [59,65]. This will promote the development of BCAs in the rhizosphere, including the creation of biofilms that prevent pathogens from colonizing the roots, the secretion of essential micronutrients such as siderophores, and an effective micronutrient absorption system compared with pests [65,66]. Indirect strategies involve promoting plant defensive responses, plant development, and soil fertilization to induce resistance. A plant’s systemic resistance, which develops structural barriers and induces the host to establish several biochemical and molecular defenses, can be initiated by BCAs [41,67]. The defense must be indicated by phytoalexins, phytohormones, and protective enzymes such as phenylalanine, chitinase, ammonia-lyase, phenolic compounds, and PR proteins [1,5]. Here, an effort has been made to summarize the important information previously documented in the literature and the current status of research related to biocontrol mechanisms for crop diseases.

### 4.1. Microbial Natural Products: A Potential Weapon in the Agriculture Sector

Microbial compounds are natural products with a powerful potential to control plant diseases. Different types of antimicrobial compounds have been isolated from beneficial bacteria and fungi [11,12]. The important natural products isolated from biocontrol microbes used for crop disease prevention are described in detail below. 

### 4.2. Bacteria as a Valuable Source of Natural Products

Beneficial bacteria produce a diverse range of natural products to efficiently manage various plant diseases. These antimicrobials produced by bacteria (2900), fungi (4900), and actinomycetes (8700 distinct antibiotics) include lipopeptides, such as iturin, surfactin, and fengycin [1,68]. *Pseudomonas* species have also been reported to produce a variety of natural products, including extracellular enzymes such as cellulase, chitinase, proteases, and beta-glucanase, as well as other antimicrobial compounds such as phenazines, siderophores, cyanide, and 2,4-diacetyl phloroglucinol, to control many plant diseases [69]. Members of the fengycin and iturin families are mainly responsible for controlling many fungal plant diseases. The hyphae and conidia of various fungal pathogens, including *F. graminearum* and *Monilinia fructicola*, have been observed to be damaged by these lipopeptides [70,71]. However, iturins and fengycins have also infrequently been found to possess an antibacterial activity against *Xanthomonas campestris* and *Pectobacterium carotovorum* [72], *X. axonopodis* pv. *vesicatoria* [73], or *R. solanacearum* [74]. Other biologically active substances produced by *Bacillus* species include mycosubtilin, subtilisin, sublancin, bacilysin, chlorotetain, mycobacillin, rhizocticins, bacillaene, and difficidin, all of which are useful in controlling various plant diseases [5,12].

The volatile organic compounds (VOCs) produced by bacteria have been documented to promote plant growth and reduce the severity of crop diseases. Bacterial VOCs are less toxic to human health and are more cost-effective as well; therefore, the identification of different VOCs with a broad-spectrum antagonistic potential attracts significant attention [2,4,75]. VOCs including alcohol, aldehydes, ketones, hydrocarbons, acids, and terpenes are responsible for controlling multiple plant pathogens [37,40]. Many studies have been conducted on the antimicrobial properties of VOCs against *S. sclerotiorum*, *B. cinerea*, *A. solani*, and *M. fructicola* [4,76]. In short, all bacterial natural products are useful in controlling various crop diseases, maintaining soil fertility, and regulating rhizosphere microbes for plant health.

### 4.3. Fungal Natural Products for Crop Disease Prevention

Fungi produce various substances that allow them to endure adverse environments. Fungal-based compounds protect agriculturally important crops from different pathogens [43]. The biocontrol potential of fungi is due to their large source of bioactive compounds and their capability to combat numerous crop diseases. Different types of natural products produced by fungi, including antibiotics, polyketides, non-ribosomal peptides, aromatic compounds, and heterocyclic metabolites, are shown in Figure 1. Numerous chemicals produced by *Trichoderma* spp. are also used as biocontrol agents in controlling many crop diseases [49,56]. *Trichoderma* spp. and *Gliocladium virens* are known to produce numerous antifungal compounds, such as gliovirin, viridiol, valinotrocin, viridin, gliotoxin, and heptelidic acid [77]. *T. harzianum* strains T22 and TC39 have been described as producing antibiotics such as 1-hydroxy-3-methylanthraquinone, azaphilone, harzianopyridone, and harzianolide. These natural products can suppress the growth of plant pathogens such as *Botrytis cinerea, Rhizoctonia solani, Leptosphaeria maculans*, *Pythium ultimum,* and *Phytophthora cinnamomin* [78]. Different types of secondary compounds have been isolated and identified from fungi using various methods, including column chromatography, high-performance liquid chromatography (HPLC), liquid chromatography−mass spectrometry (LC-MS), and gas chromatography−mass spectrometry (GC-MS) [1,5]. The three strains of *Chaetomium globosum,* i.e., Cg-7, Cg-6, and Cg-5, produced Chaetoglobosin, an antibiotic in their culture filtrate that is used to reduce post-harvest diseases in numerous fruits [79]. The biocontrol of nematodes and plant diseases has so far been associated with *Trichoderma.* Many species be particularly effective as biocontrol agents, including *T. polysporum*, *T. harzianum*, *T. gamsii*, *T. atroviride,* and *T. viride* [80].

A crucial element of soil−plant systems is the large variety of fungal communities, which interact actively in the rhizospheric and endophytic regions [50]. The rhizosphere is the area around a plant root that is inhabited by a unique population of microorganisms, while the endospheric refers to the intracellular areas of plant tissues that are inhabited by microbial endophytes without harming the host plants [4,81]. The interaction between plants and fungi in the rhizospheric and endophytic regions is very important for growth promotion and disease resistance against various pathogens [5,24]. Mycorrhizal species of root-inhabiting fungi make up a significant portion of the rhizosphere’s fungus with a great impact on plant life, including plant nutrition and growth, and provide tolerance to biotic stress [82]. Hence, these fungi can be utilized as eco-friendly biofertilizers, bioinoculants, and BCAs in place of chemical pesticides [49].

### 4.4. Competition of Biocontrol Agents with Other Rhizosphere Microbes

Many microorganisms residing in soil interact with plants in a complex manner. These microbes coexist in the rhizosphere and compete for food and space. In the rhizosphere, pathogenic and non-pathogenic microbes compete for food and other resources [3,83]. These favorable interactions between plants and microbes occur regularly in the rhizosphere, promoting plant growth and overcoming biotic and abiotic stresses [2,84]. *Pseudomonas* and *Bacillus* spp. are the most common root-colonizing bacteria in various crops. Previous research has shown that *P. jessenii* RU47 efficiently colonized lettuce roots against *Rhizoctonia solani*, resulting in growth promotion and a reduction in disease severity [77]. Many studies have reported the potential of mycorrhizal fungi for efficient root colonization in plants infected with different pathogens [49]. *Trichoderma* spp. can benefit plants in several ways in terms of growth promotion and a reduction in disease severity. Numerous studies have demonstrated that root colonization by *Trichoderma harzianum* increases plant enzyme activity to establish resistance against various pathogens [49]. As a result, successful root colonization by arbuscular mycorrhizal fungi (AMF) and some strains of nonpathogenic bacteria can boost plant resilience to biotic stressors. Microbial root colonization is significantly influenced by traits of the host plants and surrounding microorganisms [24]. Plants have been proposed to actively attract soil microorganisms by releasing chemicals into the rhizosphere to encourage bacteria that are beneficial to plant growth and health [64,85]. *Arabidopsis thaliana* roots secrete malic acid, which helps *B. subtilis* colonize the roots, providing improved plant defense against *P. syringae* [86]. Roots and their exudates supply nutrients to beneficial microbes to establish root colonization against different pathogens [2,87]. In short, the study of the advantageous effects of plant-associated microbiomes, such as the stimulation of plant growth and defense against plant diseases, has been a hot topic of interest over the past few decades, although the majority of the methods used by plants to attract bacteria are still unknown. This requires a deeper understanding of the different aspects of microbes that encourage root colonization.

### 4.5. Biocontrol Agents Promote Plant Growth

Plant-growth-promoting microbes (PGPMs) help with efficient root colonization, compete with other soil microorganisms, stimulate host defense systems against pathogens, and promote plant growth through different mechanisms [6,62]. PGPMs have biocontrol agents (BCAs) called plant-growth-promoting rhizobacteria (PGPR) and plant-growth-promoting rhizofungi (PGPF) that fight crop diseases [37]. Earlier studies reported that *Bacillus*, *Pseudomonas*, *Actinobacteria*, and *Lactobacillus* have been used in different crop protection strategies [63,64]. The biocontrol strains *B. pumilis* and *B. amyloliquefaciens* displayed many important characteristics, such as siderophore production, phosphate solubilization, IAA production, and antagonistic activity toward fungal pathogens, which could improve plant growth in terms of leaf number, biomass, and shoot length under field conditions [46,65].

**Figure 2:**
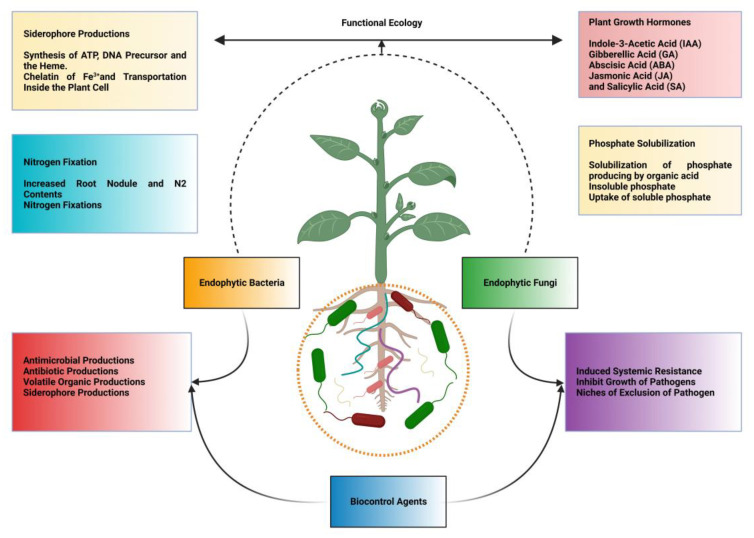
Molecular mechanism of biological control agents of bacteria and fungi on plant growth traits and plant defense resistance. BCAs, such as fungi and bacteria, can produce various types of phytohormones, nitrogen fixation, siderophore production, phosphate solubilization, and induced systemic resistance, resulting in improved plant growth and control of plant disease. BCAs help other bacteria and fungi to improve plant health. The different arrows show the different mechanism connections emitted by fungi and bacteria. The figure was created with BioRender software (https://www.biorender.com/, accessed on 13 September 2023).

Biofertilizers or microorganisms promote plant growth in many ways, including nitrogen fixation, phosphate solubilization, siderophore generation, and HCN production [31,66]. The contribution of rhizobial N-fixation to global agricultural systems is vital, ranging from 20 to 22 Tg N per year to 40 Tg N per year. [67]. Many bacterial strains increase the accessibility of Fe by producing organic acids or siderophores [31,68]. PGPR can produce auxins that strongly affect root growth and architecture [69,70]. Indole-3-acetic acid (IAA), an auxin produced by PGPR, has gained much attention in plant-growth promotion under adverse conditions [19,71]. Auxin-producing PGPRs have been shown to regulate the expression of various genes involved in plant-growth promotion and defense systems [69,72]. PGPR strains also withstand stress through the production of other important phytohormones such as gibberellins and cytokinins [73,74]. PGPR is known to produce complex secondary metabolites such as flavonoids, terpenes, and phenolic compounds, which might draw particular microbes to the rhizosphere [6,75]. Finally, we can conclude that biocontrol agents help promote plant growth through different mechanisms, which still demand detailed studies employing modern techniques used in plant−microbial interactions. The molecular mechanisms of biological control agents of bacteria and fungi on plant growth traits and plant defense resistance are briefly reported in Figure 2.

## 5. Factors Affecting Biocontrol of Plant Diseases and Selection of Potential BCAs

Despite decades of research, biological control remains relatively insignificant in managing plant health compared with chemical control. It has already been documented that biocontrol of plant pathogens under laboratory conditions (in vitro or in planta) is more effective than in open-field trials [2,5]. BCAs face greater challenges transferring from the controlled environment of a laboratory experiment to the harsh conditions encountered in the field [59]. BCA field efficacy may be equal to or better than that of synthetic pesticides; however, this could change over time and across regions. In other words, a BCA that inhibits or controls disease in the lab is unlikely to be efficient in open-field trials [85,88]. This is a result of all of the complex interactions that take place between the host, pathogen, antagonist, and environment. For instance, the host undergoes a sequence of changes or mutations that alter its physical and chemical features. The efficiency of the antagonist is also affected by pathogenic behaviors [89]. The antagonistic potential of BCA may change as a result of changes in the population and environment or from the existence of microbial colonizers in the natural environment [37,89]. *Trichoderma* is the most extensively reported fungal BCA, but its broader application has been hampered by its unpredictable nature in the field. To create and put into practice *Trichoderma*-based agricultural production and preservation methods, it is essential to fully understand how it interacts with plants, other microbes, and the environment [49,90]. The factors limiting the efficacy of BCA are given below in detail.

### 5.1. Plant Species Influence the Biocontrol Activity of BCAs

Different plant species and genotypes have a significant impact on the effectiveness of the biocontrol potential of microbes [91]. Several studies have been conducted that show the effect of plants on BCAs’ biocontrol activities. The degree of rhizosphere colonization, the production of antibiotics by BCA, and the stimulation of induced systematic resistance vary among different plant species [85]. In the rhizosphere region, the interaction between BCAs and pathogens is mostly influenced by root exudate excretion, water and mineral absorption, and other surrounding microbes [8,85]. The expression of plant genes is reported to be involved in the rhizosphere enrichment process that provides fitness to the plants by recruiting microbes from the surroundings [92]. The complexity behind the specificity of BCAs for specific hosts must be uncovered with in-depth research to control different plant diseases in a well-organized manner [13].

### 5.2. Pathogen Influence the Biocontrol Activity of BCAs

The behavior of pathogens toward BCAs is one of the most crucial factors in plant disease control. Each pathogen interacts with the host differently due to genetic variety and ecological fitness diversity [1,49]. It is crucial to emphasize that the pathogen behaves differently from the BCAs because it is both harmful and vulnerable to antagonist activity. Depending on the complexity of their mode of action, phytopathogens may display a wide range of sensitivities to BCAs (including extremely low sensitivity). In a few generations, some pathogens can adjust to the selection pressure imposed by BCAs [93]. The idea that biological control lasts longer than chemical control is a widespread misconception. According to research on pest management in fields, this assumption may not always be true. Numerous pests have developed tolerance to one or more Bt toxins, while the codling moth *Cydia pomonella* has established resistance to the *C. pomonella* granulovirus [59].

The durability of biological control of plant diseases, in contrast with pest management, has received little attention [94]. Similar to single-mode chemical fungicides, populations of pathogens could change into forms that are resistant to BCAs. This would make it less likely that BCAs will be able to reduce plant diseases for a long time. Therefore, a weak spot in the pathogen life cycle must be found as an opportunity window for successful biocontrol [93]. BCAs should enter the window of opportunity and stop the progression of pathogens. For example, the window of opportunity for some unspecialized necrotrophic pathogens is to obstruct the nutrient uptake required for growth. If BCAs are abundantly present near the pathogen spores, the depletion of local nutrients from the disease spore could hinder or stop germination [85].

### 5.3. Biocontrol Agents and Their Specific Nature

The ability of BCAs to adapt to local biotic and abiotic environmental conditions is the primary cause of their reduced field efficiency. Therefore, to completely understand this phenomenon, it is important to study the distribution pattern of BCAs in the rhizosphere [1,95]. To obtain pertinent biocontrol results, additional suitable native BCA strains should be collected and examined. The mode of action of BCAs, their selectivity against plant pathogens, and their resistance to harsh climatic conditions play a significant role in their success [2,64]. The efficacy of the biocontrol agent improves when an optimal association exists. Therefore, the application of BCAs at the right time is crucial for effective biocontrol. If the antagonist is applied before the infection becomes established, biocontrol will be effective [85,96].

Furthermore, it is crucial to understand how BCAs function to attain the best disease control effect. Numerous characteristics of fluorescent *Pseudomonas* make them excellent BCAs. These features comprise (i) the ability to efficiently colonize roots, hypocotyls, tubers, and other parts of the plant; (ii) the convenience of cultivation in the lab; (iii) the synthesis of various secondary metabolites; (iv) being able to utilize various kinds of organic compounds, which are usually found in exudates from roots and seeds; and (v) compatibility with common pesticides and biological agents [97]. Hence, future studies should focus on identifying novel strains with potent biocontrol characteristics by investigating unexplored microbial diversity. This could involve extensive sampling and screening of different habitats in order to identify microorganisms with a broad-spectrum antagonistic activity against various phytopathogens.

### 5.4. Environmental Stresses Impact on BCA Activity

The microbial makeup of the soil and its environment has a significant impact on the efficacy of biological control measures. Soil biology research should be able to figure out the unique qualities of different species, especially those that live in the rhizosphere of plants, by figuring out what each possible microbe brings to the biocontrol process. Similarly, the ecological study should investigate all biotic and abiotic factors that have a significant impact on BCAs as crucial components of plant health [59]. Many BCAs are extremely susceptible to changes in the biotic and abiotic environment. It is not always possible to transfer their efficiency from the laboratory to the field [52,98]. For instance, although many *Pseudomonas* and *Bacillus* BCAs function well in trials, they cannot effectively handle disease in a variety of field conditions [99]. Therefore, it is important to select a BCA that has stable efficacy under various environmental conditions, such as soil texture, moisture, temperature extremes, or competition [10,97]. Furthermore, knowledge about the ecological and biological components of the soil can improve the efficacy and success of BCAs in the control of plant diseases [100,101]. Finally, investigating how biocontrol strategies can contribute to enhancing crop resilience in the face of climate-change-induced stressors could be an important avenue. Understanding the interactions between biocontrol agents, pathogens, and changing environmental conditions could guide future research.

## 6. Challenges in Establishing Beneficial Microbes as BCAs

This biological control strategy was initially employed at the end of the 19th century. Displaying an array of advantages, the literature has also mentioned many limitations of this biocontrol strategy for controlling plant diseases [98,102]. As the method offers an effective alternative to chemical pesticides for controlling pests and plant diseases, there are still various challenges to overcome. These challenges are briefly discussed below.

### 6.1. The Journey of Biocontrol Agents from Lab to Field

Researchers are screening potential microbes as part of their work to create biological pesticide remedies that are very effective for controlling plant diseases in agriculture. These strains are usually chosen based on factors such as disease resistance, host range, availability, formulation, mass production, and farmer practice [1,103]. PGPR performance can be assessed by considering geographical locations, host crop species, soil types, and environmental factors. [10,89]. The growth of BCAs is typically easier to observe in controlled environments such as greenhouses. The preference of most researchers at this stage might be associated with the reliability of the environment [2,104]. The performance of BCAs in greenhouse trials can provide important theoretical and practical support for field utilization. As a result, the feasibility and efficacy of PGPR for commercial horticulture production, disease management, and field climate change conditions may be ensured [5,105].

The stability of BCAs is affected by the method of formulation, shipment, and storage environment. To achieve high levels of BCA strategy success, it is important to improve the formulation technology, extend the storage duration of the BCA product, optimize the production of selected microbial strains, and achieve large-scale application through low-cost manufacturing companies [98,106]. Many scientists are looking for a solution to increase the shelf-life of PGPR by lowering the storage temperatures and/or changing the combination of additive mixtures [107]. During the formulation and field efficacy trials, private sector partners and licensed laboratories conduct the essential environmental and human health assessments, as well as quality assurance for the ultimate commercialization of biopesticides [108]. Before utilizing biopesticides on a large scale in the field, government authorities must grant permission. Typically, the biopesticide registration and regulation portfolio includes modified versions of synthetic pesticides as well as risk evaluation. This includes ecotoxicological and toxicological tests, as well as studies into their mechanism of action and host spectrum. The majority of these requirements are difficult for authorities to achieve because producing effective biopesticides while maintaining acceptable safety and consistency standards for commercialization might be a difficult task [108,109].

### 6.2. Limited Number of Registered Biopesticides and Lack of Awareness

Despite their well-documented efficacy, BCAs now account for less than 5% of the crop protection sector’s commercial value [110]. The identification, characterization, and registration of potential microbes takes more time and requires academic−industry collaborations [102,111]. Furthermore, using natural resources to manage diseases (i.e., BCAs) raises several ethical and legal issues that might affect the biodiversity of an area [111,112]. In this situation, new BCA species and populations have been restricted from entering specified countries. However, commercial applications of PGPR in protected environments such as greenhouses are significantly easier due to the availability of more isolated and controlled environments and they have potentially less detrimental ecological impacts [77,113]. Another challenge that has risen as a result of the extensive use of PGPR-based biocontrol is regulatory concern. At present, every country has its own regulatory system, which varies widely. For example, high development costs for new commercial BCAs have been recognized as an obstacle to the BCA industry’s development in Australia [98,114]. To facilitate the registration and commercialization of novel BCAs and their products, BCA registration requires extensive cooperation among governmental institutes, universities, and industry sectors. The number of programs that provide financial and ecological benefits plays a vital role in biopesticide registration [102,115]. Hence, local usage and global marketing commercialization must fulfill international legislation. The International Biological Control Organization (IOBC) was established to bring together scholars, scientists, and professionals from a wide range of industries and fields to identify barriers and offer recommendations to overcome them [116,117].

Farmers might experience little or no financial gain when compared with chemical insecticides, which are more predictable and reliable [113,118]. Farmers are discouraged from using the biocontrol strategy if they lack of expertise and knowledge about successful technological advances. Positive initiatives, which could include community talks, training workshops, and free conferences, might increase awareness regarding the use of BCAs in certain farming areas [77,119]. PGPR-based biocontrol provides substantial advantages in terms of decreasing pesticide use in agriculture. Biocontrol management has a direct impact on farmers’ expenses and income, but it also has an indirect impact on their financial benefits due to its effect on farmland biodiversity and environmental sustainability [98]. However, farmers have not fully utilized commercial biopesticides due to a lack of information and awareness. Therefore, there is a need to strengthen the idea of biological management in the farming community to restore their trust in the use of biopesticides.

### 6.3. Biopesticides Commercialization and Legislative Procedure

Despite their growing popularity in crop disease management, biocontrol agents currently represent only 1% of agricultural control techniques, whereas synthetic-based pesticides account for 15% in the agriculture sector [120]. The commercialization of biopesticides is a multi-step procedure that faces numerous challenges. Before being licensed for commercialization, similar to synthetic pesticides, BCAs are subjected to risk evaluations [121]. European Regulation (EC) No. 1107/2009 specifies standards based on risk assessment in plant protection product marketing. European Communities (EC) Regulation No. 540/2011 specifies microbes that have been certified to be used as biological controls in Europe. As a result, commercial authorizations for biopesticides (and synthetic pesticides) can be obtained after an extensive procedure. However, the current trend of reducing the use of synthetic pesticides and simplifying the regulatory process for low-risk products may allow BCAs to be commercialized worldwide [108,109].

## 7. Biotechnological and Omics Techniques for Biocontrol Strategy Improvement

A large amount of microbial genomic data are easily accessible and have opened a new chapter for the discovery of potential BCAs [122]. Integrating advanced omics and metagenomic techniques could provide a deeper understanding of the interactions between beneficial microbes and pathogens. Studying the genomic, transcriptomic, and proteomic profiles of microbes, pathogens, and plants during biocontrol interactions could reveal key mechanisms and factors influencing disease suppression [123,124]. It is expected that by using biotechnological approaches, researchers will be able to develop potential BCAs for efficient control of multiple crop diseases [43]. Based on the information we have collected, we have done our best to summarize the recent advancements in biocontrol strategies in light of prominent emerging biotechnological and omics techniques.

### 7.1. Biotechnological Techniques Linked with Biocontrol Strategy

Using biotechnological techniques and synthetic biology provides an interesting direction for biocontrol strategy improvement. Through advanced techniques in microbial engineering, researchers could create microorganisms with strong phytopathogen suppression features, including increased synthesis of antimicrobial substances and efficient colonization of plant surfaces [125,126]. There are many examples where BCAs are successfully modified for desired traits. Using a genetic engineering tool, introducing glucanase gene to *Trichoderma,* resulted in increased resistance to diseases such as *Pythium*, *Rhizoctonia*, and *Rhizopus* [57]. Transferring a *Serratia* gene expressing the chitinase enzyme to the *Pseudomonas* endophyte also reduced *Rhizoctonia solani* disease in beans [57,127]. The biosynthetic locus phlACBDE from strain CPF-10 was cloned into a mini-Tn5 transposon, and then the chromosome of *Pseudomonas fluorescens* P32 improved tomato tolerance to *R. solanacearum* bacterial wilt and wheat resistance to *Gaeumannomyces graminis* var. *tritici* [128]. Several microorganisms associated with plants can produce plant hormones such as auxin, ethylene, and cytokinins, all of which play important roles in plant growth and disease resistance [1,2]. The pathways responsible for these hormones are engineered to express them in other species. Heterologous expression of the IAA synthesis pathway in *Bacillus* spp. significantly increased IAA production, efficiently enhancing plant growth in harsh conditions [129,130]. The banana endophytes *Kosakonia* sp. S1 and *Enterobacter* sp. E5 produced ACC deaminase, which enhanced banana plant development and improved tolerance to *Fusarium* wilt. Hence, researchers might be able to formulae a BCA that will efficiently mitigate the harsh conditions in the open field and maintain plant health [131,132]. Using tools such as CRISPR/Cas, we can insert mutations into specific sections of the genome with high precision and efficiency. In addition, mutations can be made in multiple genes at the same time, enabling the determination of the role of various genes in biocontrol. Engineered BCA could help detect plant physiological variations caused by biotic stresses, besides delivering desirable characteristics [133,134].

### 7.2. Biocontrol Strategy in the Era of Multi-Omics

**Figure 3:**
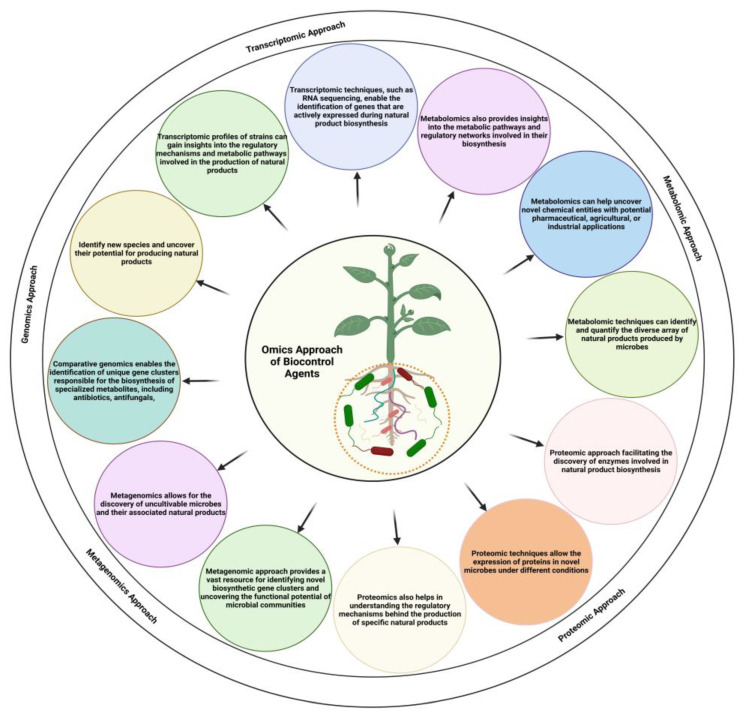
Multi-omics approaches such as genomics, metagenomics, proteomics, transcriptomics, and metabolomics need to be used to identify potential microbial strains, a source of antimicrobial products. Omics also provides insight into the complex molecular mechanisms that support plant−pathogen interactions. The picture was made with the BioRender program (https://www.biorender.com/, accessed on 13 September 2023).

An effective approach to better understanding the interactions of BCAs with the host plant, pathogen, and environment is now offered through an influx of new generations of molecular technologies [135]. The name “omics” refers to these emerging technologies that involve next-generation sequencing (NGS) technology, proteomics, metabolomics, genomics (including its derivatives pangenomics and metagenomics), and transcriptomics [126,136]. The use of diverse omics approaches could ultimately accelerate the development of BCAs against plant diseases. Omics also provide insight into the complex molecular mechanisms that support plant−pathogen interactions, aiding in the identification of functional elements in pathogens involved in disease [123,137]. The details of different Omics techniques used to improve biocontrol strategy are briefly summarized in Figure 3.

Exploring endophytes’ role in plant disease protection is an exciting area of omics research. Endophytes are microorganisms that reside within plant tissue without causing visible symptoms and are vital for plant health. At present, most studies have focused on the function of one or a few endophytes rather than complete communities [53,127]. It has been reported that *Burkholderia phytofirmans* PsJN, through various extra-cytoplasmatic functional group elements (sigma factors and group IV), assist other bacteria in monitoring changes in their environment, such as extreme temperatures, and in altering their metabolism to survive in harsh conditions. This study used a dual omics technique, applying high-throughput DNA sequencing to identify *Burkholderia*, as well as other bacteria, and conducting critical metabolomics. Microorganisms that inhabit the soil are essential components of the ecosystem, playing crucial roles in plant growth, development, defense, and responses to stress [138,139]. As a result, understanding the relationship between plants and the soil microbial community utilizing metagenomics will be useful in constructing agricultural systems for progressive sustainable agriculture production [38,137]. The use of metagenomic techniques in the study of soils inoculated with organic manures would be beneficial for developing fertilization strategies to decrease the dependence on chemical fertilizers [140].

### 7.3. Microbiome-Based Solution for Plant Disease Management

Multi-omics and next-generation sequencing (NGS) reveal enormous microbial diversity across many plant species, as well as different phytopathogen antagonistic microbes [38,123]. According to newly available data, a wide range of microorganisms effectively interact with plants in the rhizosphere region. The knowledge related to different factors that influence microbes in the rhizosphere region will be helpful for future efficient biocontrol strategies [141]. The earliest plants grown on Earth, mosses, are notable for their extraordinary microbiological diversity and high level of antagonistic potential because of their ecology [142,143]. In addition, medicinal plants have been determined to be a fascinating source of biodiversity. It has recently been discovered that archaea are a component of the plant microbiome, and their impacts on plants, as well as their biocontrol potential, remain unexplored [144,145]. Hence, the use of different omics techniques will enable us to identify different untapped microbes from different regions that can be formulated further to improve the biocontrol strategy [146].

Furthermore, to maintain ecosystem diversity and health, integrated breeding and biocontrol measures are essential [147]. These systematic approaches are essential to prevent further biodiversity loss and encourage long-term farming practices. Crop breeding has been highlighted as a major force in natural evolution. Currently, the breeding strategy is aimed at controlling diseases, increasing yield, and various growth features; however, in the past, only a few plant phenotypes were randomly selected, and changes in the plant microbiome were ignored [87,148]. Although the use of PGP bacteria in the field appears to be a viable strategy for sustainable agriculture, efficacy has proven inconsistent, probably due to variations in environmental conditions, poor microbial colonization, and low long-term stability in the rhizosphere [94,149]. These limitations could be minimized by genetic or genome engineering of active root colonizers or improved colonization with large phyto-microbiome subpopulations [132]. Soon, consortiums of microbes and biocontrol products may be utilized to improve biodiversity associated with crops via microbiome engineering to accomplish specific microbiome outcomes.

### 7.4. Microbiome Engineering: A Shining Approach in Biocontrol Strategy

It has already been documented that agriculture could benefit significantly from microbiome engineering. Therefore, a modified microbiome with essential features is required for crop disease management [132]. Plant-associated beneficial microorganisms have enormous potential for commercial and sustainable agriculture. Plant microbiome engineering can be achieved in two ways: bottom-up methods involve isolating, altering, and reviving certain microbes, whereas top-down approaches involve synthetic ecology, which includes horizontal gene transfer to a variety of hosts in situ and then phenotyping the microbiome [112,132]. The recent advancements in genome engineering tools, computational tools, genome-wide functional tools, and meta-omic tools can improve our ability to design microbes for biocontrol and biofertilization, as well as increase agricultural productivity and yield [126]. Current advances also report some devices that permit the evaluation of engineered microbiome function before field studies. For instance, lithographic 3D printing has made it possible to create small microscopic containers for arranging several bacterial species in virtually any 3D geometry [132]. It will enable us to study their interactions at a microscopic level and compare them with those of native strains. The “tracking root interactions system” (TRIS), a microfluidic device developed to track root−bacteria interactions, is also beneficial for studying the engineered microbiome [150]. From a practical point of view, it would be extremely helpful to develop microbiomes that are long-lasting, stress-resistant, and capable of increasing agricultural output. Finally, microbiome bioengineering is a fascinating choice for improving plant health. This approach, still in the early stages, will provide enormous benefits to biocontrol methods in the future [126].

## 8. Research Gaps and Future Direction

Biocontrol of plant diseases is an emerging strategy that still leaves many gaps for future research work. The journey of biocontrol agents from lab to field requires multiple efforts to reach a high peak. Further research could focus on discovering novel strains with potent biocontrol features by exploring untapped microbial diversity. This could involve extensive sampling and screening of various habitats to discover microbes with broad-spectrum antagonistic activity against different plant pathogens. By integrating modern omics and metagenomic approaches, researchers may be able to gain advanced knowledge of the relationships between beneficial bacteria and pathogens. The genomic, transcriptomic, and proteomic profiles of microorganisms and plants during biocontrol interactions may reveal critical mechanisms and factors affecting disease suppression. Using synthetic biology principles to design beneficial bacteria with better biocontrol capabilities could be a future avenue. Researchers could develop microorganisms with more disease-fighting properties, such as improved antimicrobial substance production or increased colonization of plant surfaces. In addition, nanotechnology-based biopesticides offer an environmentally responsible and efficient pest management. Biopesticides, in particular nano-biopesticides, have the potential to revolutionize global agriculture in terms of protecting crops for sustainable agriculture. 

Modification of the plant microbiome to increase the growth and activity of beneficial microbes could be a promising approach. Upcoming research could look into fruitful ways to manipulate the composition and function of the plant microbiome to produce an environment suitable for biocontrol. The performance of BCAs in the greenhouse experiment can provide significant theoretical and practical support for field utilization. Future research could focus on large-scale trials to evaluate the practical effectiveness of biocontrol agents in different agroecosystems and under varying environmental conditions. Biocontrol could be incorporated into complete IPM programs that include a variety of techniques, such as cultural practices, resistant crop varieties, and biological control agents. Such integrated approaches, when developed and optimized, could lead to more sustainable and reliable crop protection systems.

Future research should also look into the economic feasibility and societal consequences of utilizing biocontrol technologies on a broader scale. Analyzing the cost-effectiveness and potential benefits for farmers and the agriculture sector as a whole would be useful. It is critical for the widespread use of biocontrol strategies to spread knowledge about them among farmers, extension services, and policymakers. More efforts could concentrate on building educational programs and outreach campaigns to improve awareness and make biocontrol measures more practical. Considering biocontrol’s potential to minimize dependency on chemical pesticides, further study could help build regulatory frameworks and policies that support the safe and effective use of biocontrol agents in agriculture. Finally, investigating how biocontrol methods can help enhance crop resilience in the face of climate-change-induced challenges could be a promising direction. Future studies could be motivated by knowledge of the relationship between biocontrol agents, diseases, and changing environmental conditions. Researchers can improve the subject of biocontrol to create more sustainable and effective crop disease management systems by focusing on the research gaps mentioned above.

## 9. Conclusions

The application of beneficial microbes to control crop diseases is a safe and eco-friendly strategy that has attracted the attention of many scientists in the crop protection sector. This review addresses the importance of biocontrol strategies in preventing important diseases in crops. Moreover, for effective biological control strategies in the future, more research studies must be conducted on some less developed aspects, including the development of potential biocontrol agents and the use of biotechnology in conjunction with “omics” techniques to improve biocontrol strategies. Targeted and predictive biocontrol methods can be developed through a comprehensive strategy and the incorporation of microbiome-based solutions. In addition, a combination of breeding and biocontrol measures is needed to preserve ecosystem diversity and health. Thus, integrated approaches are needed to protect biodiversity from further decline and to maintain sustainable agricultural practices. Therefore, this review will be more helpful to researchers studying plant−microbe interactions as it provides sufficient knowledge on the current status and needs for the further development of biocontrol strategies to ensure a sustainable plant disease management system.

## Figures and Tables

**Table 1:** Various bacterial and fungal biocontrol agents against plant pathogens.

Plant Species	Biocontrol Agents	Pathogens	Mode of Action	Ref.
	Bacterial strains			
Citrus fruit	*Bacillus megaterium*	Blue mold	In vitro antagonistic activity against post-harvest disease	[16]
Wheat	*Bacillus subtilis* 26DCryChS	*Stagonospora nodorum* Berk	Antimicrobial metabolites (surfactants showed antifungal activity against *S. nodorum* disease)	[17]
*Brassica campestris* L	*Bacillus thuringiensis*	*Sclerotinia sclerotiorum*	Suppressing *S. sclerotiorum* growth by inducing systemic resistance	[18]
Cotton/black root rot	*Paenibacillus alvei* K-165	*Thielaviopsis basicola*	K-165 inhibited *T. basicola* growth *invitro* through antibiosis and significantly reduced root discoloration and hypocotyl lesions on cotton seedlings	[6]
Tomato and Soybean	*Bacillus velezensis* DMW1	*Phytophthora sojae* and *Ralstonia solanacearum*	Antimicrobial metabolites (fengycin, iturin, and bacillomycin) demonstrated antagonistic activity in vitro and in pot experiments	[19]
Rice	*Bacillus atrophaeus* FA12 and *B. cabrialesii* FA26	*Xanthomonas oryzae* pv. *oryzae* (Xoo)	In vitro, antagonistic activity against various fungal pathogens significantly reduced Xoo lesions in greenhouse conditions	[20]
Rice	*Bacillus thuringiensis* GBAC46	*Aphelenchoides besseyi*	In vitro antagonistic activity through various proteins (Cry31Aa, Cry73Aa, and Cry40ORF) and in greenhouse conditions	[21]
Maize	*Pseudomonas protegens* Pf-5	*Pantoea ananatis* DZ-12	Antimicrobial pyoluteorin showed strong antagonistic activity against *P. ananatis* in vitro and in vivo	[22]
Wheat and Maize	*Bacillus Subtilis* ATCC6633	*Fusarium graminearum* and *Fusarium verticillioides*	Antimicrobial mycosubtilin showed a strong antagonistic activity against *F. graminearum* and *F. verticillioides* in vitro and in vivo	[23]
Tomato	*Bacillus atrophaeus* GBSC56	*Meloidogyne incognita*	Antimicrobial VOCs showed nematicidal activity and also produced ROS in nematodes	[2]
Rice	*Bacillus* spp. GBSC56, SYST2, and FZB42	*Aphelenchoides besseyi*	Antimicrobial VOCs of *Bacillus* spp. showed the strongest nematicidal activity and accumulated ROS as well as promoted rice growth	[24]
Soybean and Rice	*Pseudomonas parafulva* JBCS1880	*Xanthomonas axonopodis* pv. *glycines*, and *Burkholderia glumae*	Strong antagonism and antibacterial activity against *Xanthomonas axonopodis* pv. *glycines* and *Burkholderia glumae*	[25]
Rice	*Pseudomonas putida* BP25	*Magnaporthe oryzae*	BP25 showed strong biocontrol activity against blasts caused by *M. oryzae*	[26]
Pepper	*Bacillus licheniformis* BL06	*Phytophthora capsici*	BL06 effectively reduced pepper *Phytophthora* blight severity in vitro and pot experiments	[27]
Wheat	*Bacillus atrophaeus strain* TS1	*Fusarium graminearum*	TS1 was found as a potential biocontrol agent to inhibit *F. graminearum* under low temperatures	[5]
Tomato	*Bacillus amyloliquefaciens* FZB42	*Sclerotinia sclerotiorum*	Antimicrobial potential (fengycin-induced systemic resistance in tomatoes against *S. sclerotiorum*)	[1]
Rape Seed and Tabaco	*Bacillus amyloliquefaciens* EZ1509	*Sclerotinia sclerotiorum*	*Bacillus* strain EZ1509 showed a strong antifungal activity against *S. sclerotiorum* and also led to the development of new biopesticides	[12]
Tomato	*Streptomyces* sp. AN090126	*Ralstonia solanacearum* and *Xanthomonas euvesicatoria*	*Streptomyces* sp. AN090126 can combine with antibiotics effectively control different bacterial plant diseases	[28]
	**Fungal strains**			
Tomato	*Paecilomyces lilacinus*	*Meloidogyne javanica*	*P. lilacinum* is used as a biocontrol agent to control *M. incognita* and as a better alternative against chemical nematicides	[29]
Pineapple	*Purpureocillium lilacinum*	*Meloidogyne javanica*	The application of *P. lilacinum* significantly reduced nematode egg and egg mass production, reducing root galling damage in pineapple	[30]
Onion	*Trichoderma asperellum*	*Sclerotium cepivorum*	*T. asperellum* BCC1 exerts efficient biocontrol against *S. cepivorum* and activates onion systemic defenses against *S. cepivorum* under greenhouse conditions	[31]
Okra	*Trichoderma virens*	*Meloidogyne incognita*	*T. virens* observed a reduction in second-stage juveniles’ hatching periods tested in vitro	[32]
Carrot	*Pochonia chlamydosporia*	*Meloidogyne incognita*	*P. chlamydosporia* reduced nematode galls and also decreased juvenile 2 nematodes in vitro and pot experiment methods	[33]
Mango	*Trichoderma asperellum T8a*	*Colletrotrichum gloeosporiodes*	*T. asperellum* T8a plays a role in biological control against *C. gloeosporioides* and controlling anthracnose disease in mangoes	[34]
Beans	*Trichoderma asperellum*	*Sclerotinia sclerotiorum*	*T. asperellum* the reduced disease severity index and antagonistic activity against *S. sclerotiorum* in field trials of beans	[35]
Cabbage	*Trichoderma hamatum*	*Sclerotinia sclerotiorum*	*T. hamatum* LU593 reduced apothecial production, decreased disease severity index, and could potentially control *S. sclerotiorum* disease in cabbage	[36]

## Data Availability

Not applicable.
